# Air exposure test in *Colossoma macropomum* juveniles maintained in water at suboptimal temperature: Physiological, metabolic and oxidative stress responses

**DOI:** 10.1111/jfb.70450

**Published:** 2026-04-08

**Authors:** Gisele Cristina Favero, Luanna do Carmo Neves, Suzane Lilian Beier, Hugo Napoleão Pereira da Silva, Juan Lameira Dornelles, Bernardo Baldisserotto, Glauber David Almeida Palheta, Nuno Filipe Alves Correia de Melo, Ronald Kennedy Luz

**Affiliations:** ^1^ Laboratório de Aquacultura, Departamento de Zootecnia, Escola de Veterinária Universidade Federal de Minas Gerais Belo Horizonte Brazil; ^2^ Departamento de Clínica e Cirurgia Veterinárias, Escola de Veterinária Universidade Federal de Minas Gerais Belo Horizonte Brazil; ^3^ Laboratório de Fisiologia de Peixes, Departamento de Fisiologia e Farmacologia Universidade Federal de Santa Maria Santa Maria Brazil; ^4^ Programa de Pós‐graduação em Aquicultura e Recursos Aquáticos Tropicais, Instituto Socioambiental e dos Recursos Hídricos Universidade Federal Rural da Amazônia Belém Brazil

**Keywords:** air exposure, oxidative stress, recovery, tambaqui, water temperature

## Abstract

This study evaluated the physiological, metabolic and oxidative responses of *Colossoma macropomum* subjected to air exposure when maintained in water at suboptimal temperature (23°C). A total of 54 fish (81.33 ± 11.90 g) were distributed into nine tanks (6 fish per tank). For the air exposure test, 24 animals were exposed for 30 min and another 24 animals for 60 min. Six fish from each group were sampled immediately after exposure and at 1, 24 and 48 h post‐exposure for blood collection; then they were killed, and the brain, gills, liver and kidneys were obtained for oxidative stress analyses. Air exposure and collection time led to changes in haematological, biochemical, gasometric and electrolytic parameters as well as those in oxidative stress in tissues. The fish modified their physiological parameters when subjected to stress by air exposure for 30 or 60 min, reestablishing baseline conditions at 48 h after air exposure, even when maintained in water at suboptimal temperature.

## 1 INTRODUCTION

Air exposure tests have been carried out in studies of different species of fish with exposure times varying from 30 s to 105 min (Faria et al., 2021; Hur et al., 2019; Mattioli et al., 2019; Neves et al., 2022; Pereira‐da‐Silva & Oliveira, 2016; Silva et al., 2012; Silva et al., 2021; Skrzynska et al., 2018). These tests have been performed with the aim of evaluating physiological disorders, as such disorders have implications for plasma glucose and/or lactate levels (Abreu & Urbinati, 2006; Inoue et al., 2008; Mattioli et al., 2019; Neves et al., 2022; Pereira‐da‐Silva & Oliveira, 2016; Skrzynska et al., 2018; Suski et al., 2007; Trushenski et al., 2010), haematological responses (Abreu & Urbinati, 2006; Hur et al., 2019; Neves et al., 2022; Silva et al., 2012; Suski et al., 2007), blood gas and ion concentrations (Ferguson & Tufts, 1992; Neves et al., 2022; Silva et al., 2012; Suski et al., 2007), growth performance (Silva et al., 2021) and oxidative status (Faria et al., 2021; Paital, 2013) of fish.

Oxidative stress results from an imbalance between the production of reactive oxygen species (e.g. hydrogen peroxide and free radicals) and the antioxidant defence system (Birnie‐Gauvin et al., 2017) and has been measured in different fish tissues, such as brain (Dolci et al., 2013; Ekabaram et al., 2014; Lushchak & Bagnyukova, 2006; Lushchak & Bagnyukova, 2007; Piner et al., 2007; Taylor et al., 2015), gills (Ale et al., 2018; Chen et al., 2012), liver (Baldissera et al., 2019; Faria et al., 2021; Lushchak et al., 2001; Lushchak & Bagnyukova, 2006; Lushchak & Bagnyukova, 2007; Ming et al., 2019; Taylor et al., 2015) and kidney (Dolci et al., 2013; Lushchak & Bagnyukova, 2006). Faria et al. (2021) investigated the influence of common stressors in aquaculture, such as exposure to air and chase, on the oxidant and antioxidant systems in fish and found that both stressors promoted increased superoxide dismutase (SOD) enzyme activity and reduced activities of CAT and reduced glutathione (GSH)‐peroxidase in the liver of juveniles of *Piaractus mesopotamicus*, a native South American round‐shaped fish, as are *Colossoma macropomum* and *Piaractus brachypomus* (Valenti et al., 2021).

*C. macropomum*, or tambaqui, is a native species of the Amazon basin that is widely distributed in tropical regions of South America (Fernandes et al., 2018; Prado‐Lima & Val, 2016). It is one of the most produced species in Brazil, second only to tilapia (Valenti et al., 2021). According to IBGE (2026), tambaqui production reached 113,000 t in 2024. The species is tolerant of changes in water quality parameters, such as temperature, and is resistant to hypoxia (Neves et al., 2020; Prado‐Lima & Val, 2016; Val & Almeida‐Val, 1995; Wood et al., 2018). Furthermore, studies such as Silva et al. (2021) showed that tambaqui juveniles with a final weight of 7.0 g survived for up to 75 min when exposed to air.

In its natural environment, tambaqui lives in water temperatures ranging from 25 to 34°C (Araújo‐Lima & Gomes, 2005; Fernandes et al., 2018). In the study by Neves et al. (2022), tambaqui juveniles subjected to 28°C of water temperature were able to restore their physiological homeostasis within 48 h after exposure to air (30 and 60 min). However, Amanajás and Val (2023) state that tambaqui do not tolerate temperatures below 23°C.

Due to the growing demand for tambaqui in Brazil and other countries, it is essential that its production occurs in different regions of the country, where the average water temperature is lower compared to the temperature of its original habitat. According to Fernandes et al. (2018), although tambaqui production is possible throughout the national territory, producers are discouraged from rearing it in colder regions, such as the south and southeast, due to high mortality rates during the winter months. Therefore, it is necessary to expand knowledge of how juvenile tambaqui behave physiologically when reared at suboptimal temperatures and how this species reacts to suboptimal temperatures and when subjected to stress by air exposure. The present study aimed to assess the physiological responses, in addition to oxidative responses, in tambaqui juveniles maintained in water at suboptimal temperature (23°C) and subjected to stress by air exposure and subsequent recovery.

## 2 MATERIALS AND METHODS

The experimental trial was carried out at the Laboratory of Aquaculture of the Federal University of Minas Gerais (UFMG), Belo Horizonte, Minas Gerais, Brazil. The Ethics Committee for the Use of Animals (CEUA) of UFMG approved the experimental procedures under protocol number 183/2019.

### 2.1 Fish and experimental conditions

A total of 54 *C. macropomum* juveniles (81.33 ± 11.90 g and 17.43 ± 8.70 cm) were distributed into nine 30‐L tanks (6 animals per tank) in a re‐circulating aquaculture system with constant individual aeration.

All fish were exposed to the same environmental and management conditions during a total acclimation period of 15 days, with a water temperature of 23.08 ± 0.17°C and dissolved oxygen concentration of 7.11 ± 0.09 mg L^−1^ (measured using a digital oximeter, Ecosense DO200A multiparameter probe); a pH value of 7.08 ± 0.43, salinity of 0.12 ± 0.00 g L^−1^ salt and conductivity of 0.23 ± 0.005 mS cm^−1^ (measured using a multiparameter analyzer, Hanna, Combo pH and EC Waterproof model HI98130 high‐range Sigma‐Aldrich Labware); and a total ammonia concentration of 0.22 ± 0.08 mg L^−1^ (measured using a LabconTest commercial kit). During the entire acclimatization period, the fish were fed a commercial extruded diet (Laguna 4 mm, 32% crude protein, 5% ether extract and 5% crude fibre), twice a day (8:00 AM and 4:00 PM), until apparent satiety.

### 2.2 Air exposure test

After the acclimation period, fish were fasted for 24 h, and on the following day, six animals were taken for blood sampling and killed by desensitization on ice and decapitation to establish baseline levels. Subsequently, 24 animals were subjected to air exposure for 30 min, whereas another 24 were subjected to air exposure for 60 min. Air exposure was performed by carefully removing fish from their tanks using a net, drying them with a towel and placing them individually in water‐free plastic boxes (20 × 30 cm) for the 30 or 60 min.

Six fish from each group were collected immediately after air exposure (0 h AE) and at 1 (1 h AE), 24 (24 h AE) and 48 h (48 h AE) after air exposure for blood sampling and subsequent determination of haematological, biochemical and gasometric variables. The same animals were subsequently killed in the same way as the baseline group, and brain, gills, liver and kidneys were removed and stored in a freezer at −80°C for subsequent oxidative stress analysis. Survival was monitored throughout the experimental period.

### 2.3 Blood analysis

For blood analysis, fish were held in a damp cloth, and blood was collected by caudal venipuncture. Two blood samples were taken from each animal. First, 300 μL of blood was collected using heparinized syringes for later determination of the following gasometric parameters: pH (hydrogen potential), PvCO_2_ (partial pressure of venous carbon dioxide, mmHg), PvO_2_ (partial pressure of venous oxygen, mmHg), sO_2_ (oxygen saturation, %), tO_2_ (oxygen ratio, mmol L^−1^), HCO_3_^−^ (bicarbonate, mmol L^−1^), StHCO_3_^−^ (standard bicarbonate, mmol L^−1^), BE (base excess, mmol L^−1^) and lactate (mmol L^−1^), in addition to electrolytic parameters such as concentrations of K^+^ (mmol L^−1^), Na^+^ (mmol L^−1^), Cl^−^ (mmol L^−1^) and Ca^2+^ (mmol L^−1^). All gasometric data were determined using a blood gas metre (ABL800 BASIC analyzer‐Radiometer), with water temperature and oxygen saturation corrected, according to experimental treatment, by the equipment.

Subsequently, 1 mL of blood was collected and stored in microtubes containing sodium heparin (10%) for the determination of haematocrit, haemoglobin and plasma protein. Haematocrit (%) was assessed using capillary tubes filled two thirds with previously homogenized blood. The tubes were centrifuged for 10 min at 10,867*g* (Micro SPIN 1000) and then read in a microhaematocrit using an appropriate ruler.

Plasma protein (g dL^−1^) was measured using a manual refractometer (RHC 200‐ATC, Huake Instrument Co., Ltd; www.instrument-china.com), after breaking the microhaematocrit tube just above the white blood cell layer, and haemoglobin concentrations (g dL^−1^) were determined using a colorimetric kit (reference no.; K023‐1 QUIBASA Química Basica Ltd, Belo Horizonte, Minas Gerais, Brazil, Bioclin: www.bioclin.com) through reaction with cyanomethaemoglobin. The percentage of mean corpuscular haemoglobin concentration (MCHC) was determined by the relationship between haemoglobin concentration and haematocrit.

The remaining whole blood was centrifuged at 10,867*g* for 10 min (Micro SPIN 1000) for plasma separation and glucose determination (mg dL^−1^) using the glucose oxidase method (GOD Trinder) (reference no.: K082‐2), monoreagent triglycerides (mg dL^−1^) by the Trinder reaction (reference no.: K117) and cholesterol (mg dL^−1^) by the Trinder enzymatic method using a commercial kit (reference no.: K083‐3). The enzymes alanine aminotransferase (ALT, UI, reference no.: K035‐1) and aspartate aminotransferase (AST, UI, reference no.: K034‐1) were determined using the Reitmamm and Frankel method. All tests were analysed using commercial colourimetric kits (Bioclin‐QUIBASA Química Basica Ltd), and the reading was obtained using a spectrophotometer (Biocrom Libra S22, analiticaweb.com.br).

### 2.4 Oxidative status

Brain, gills, liver and kidneys were sampled and homogenized in 0.3 M phosphate buffer +140 mM KCl (pH 7.4, 1:10) to determine the levels of GSH, SOD and thiobarbituric acid reactive substances (TBARS). GSH concentration was determined according to Sedlak and Lindsay (1968) by reaction with 5,5′‐dithiobis‐2‐nitrobenzoic acid, with cysteine as a standard. SOD activity was determined based on the auto‐oxidation principle of pyrogallol, which is inhibited in the presence of SOD. The optical density change was determined kinetically for 2 min at 10‐s intervals at 420 nm, according to Beutler (1984). Results are expressed as units per milligram of protein (U mg protein^−1^).

TBARS reaction was determined as described by Ohkawa et al. (1979), with a malondialdehyde (MDA) solution as a reference standard. TBARS levels were determined by absorbance at 532 nm and were expressed as MDA equivalent nmol MDA g tissue^−1^.

### 2.5 Statistical analysis

The experiment was carried out using a completely randomized design. Data were evaluated for normality using the Shapiro–Wilk test and homogeneity of variances using the Brown–Forsythe test; then two‐way analysis of variance (ANOVA) and Duncan's test were conducted to compare the means to evaluate air exposure durations (30 and 60 min) and collection times (0 h AE, 1 h AE, 24 h AE and 48 h AE).

## 3 RESULTS

### 3.1 Haematological and blood biochemical parameters

No mortality was observed for 48 h after air exposure. Haematocrit and haemoglobin differed significantly among collection times, in which 0 and 1 h AE had higher means for both parameters compared to 24 and 48 h AE. No significant differences were observed for MCHC (Table 1).

**TABLE 1:** Values (mean ± standard error of the mean) of haematological parameters for air exposure time (30 and 60 min) and blood collection time (0 h AE, 1 h AE, 24 h AE and 48 h AE) for *Colossoma macropomum* maintained in water at suboptimal temperature (23°C).

	Haematocrit (%)	HB (g dL^−1^)	MCHC (%)
Means for basal			
Basal	23.20 ± 2.67	7.29 ± 0.44	29.66 ± 2.80
Means of exposure (*E*)			
30 min	22.29 ± 0.49	6.03 ± 0.14	27.26 ± 0.77
60 min	22.64 ± 0.50	6.32 ± 0.16	28.52 ± 0.81
Means of collections (*C*)			
0 h AE	25.75 ± 0.69^a^	6.77 ± 0.20^a^	26.46 ± 1.08
1 h AE	23.87 ± 0.72^a^	6.78 ± 0.22^a^	30.32 ± 1.21
24 h AE	20.83 ± 0.69^b^	5.74 ± 0.20^b^	27.79 ± 1.08
48 h AE	19.42 ± 0.69^b^	5.41 ± 0.21^b^	26.99 ± 1.08
Means for *E* × *C*			
30 0 h AE	25.83 ± 0.97	7.01 ± 0.28	27.38 ± 1.53
60 0 h AE	25.67 ± 0.97	6.53 ± 0.28	29.12 ± 1.53
30 1 h AE	22.33 ± 0.97	6.48 ± 0.28	25.03 ± 1.53
60 1 h AE	25.40 ± 1.07	7.07 ± 0.35	27.49 ± 1.53
30 24 h AE	21.50 ± 0.97	5.36 ± 0.28	25.54 ± 1.53
60 24 h AE	20.17 ± 0.97	6.13 ± 0.28	31.52 ± 1.88
30 48 h AE	19.50 ± 0.97	5.27 ± 0.28	30.56 ± 1.53
60 48 h AE	19.33 ± 0.97	5.55 ± 0.31	26.48 ± 1.53
*p*‐Value exposure (*E*)	0.618	0.177	0.263
*p*‐Value collection (*C*)	<0.001	<0.001	0.766
*p*‐Value interaction (*E* × *C*)	0.169	0.154	0.812

*Note*: Means followed by different letters in a column differ by Duncan's test (*p* < 0.05). Asterisk indicates significant difference compared to basal using Student's *t*‐test (*p* < 0.05). 0 h AE, immediately after exposure; 1 h AE, 1 h after exposure; 24 h AE, 24 h after exposure; 48 h AE, 48 h after exposure. Abbreviations: HB, haemoglobin; MCHC, mean corpuscular haemoglobin concentration.

Table 2 presents the biochemical parameters of blood. An effect of collection time and an interaction between factors for lactate were observed. Fish of both air exposure durations had higher lactate levels compared to baseline up to 1 h AE, with those exposed for 60 min exhibiting the lowest lactate levels at 24 h AE. Effects of exposure duration and collection time were observed for glucose with a significant interaction between factors; the highest glucose value was observed immediately after fish were exposed to air for 60 min. Fish of both air exposure durations had higher glucose values than baseline up to 24 h AE.

**TABLE 2:** Values (mean ± standard error of the mean) of biochemical parameters for air exposure time (30 and 60 min) and blood collection time (0 h AE, 1 h AE, 24 h AE and 48 h AE) for *Colossoma macropomum* maintained in water at suboptimal temperature (23°C).

	Lactate (mmol L^−1^)	GLU (mg dL^−1^)	TGR (mg dL^−1^)	CLR (mg dL^−1^)	PPT (g dL^−1^)	ALT (UI)	AST (UI)
Basal	2.32 ± 0.56	50.81 ± 1.10	173.10 ± 31.19	84.46 ± 4.44	5.22 ± 0.15	4.85 ± 0.34	20.32 ± 4.11
Means of exposure (*E*)							
30 min	3.76 ± 0.18	80.54 ± 5.38^b^	201.18 ± 19.72	76.62 ± 2.10	5.06 ± 0.31	10.32 ± 0.50	31.75 ± 2.83
60 min	3.59 ± 0.21	99.19 ± 8.45^a^	204.34 ± 19.05	82.67 ± 2.32	5.04 ± 0.17	9.94 ± 0.56	29.29 ± 2.57
Means of collections (*C*)							
0 h AE	5.63 ± 0.20^a^	128.08 ± 4.34^a^	161.20 ± 29.39^b^	80.07 ± 3.19	5.05 ± 0.08	7.11 ± 0.93^b^	29.71 ± 3.75
1 h AE	6.27 ± 0.43^a^	108.44 ± 4.57^b^	156.10 ± 26.29^b^	77.56 ± 3.63	5.15 ± 0.08	10.23 ± 0.70^a^	36.34 ± 4.44
24 h AE	1.29 ± 0.20^b^	67.20 ± 4.57^c^	143.83 ± 27.58^b^	82.76 ± 2.88	4.97 ± 0.07	11.78 ± 0.67^a^	25.02 ± 3.68
48 h AE	1.51 ± 0.21^b^	55.73 ± 4.34^c^	350.57 ± 26.29^a^	78.19 ± 2.74	5.03 ± 0.07	11.39 ± 0.67^a^	31.00 ± 3.36
Means for *E* × *C*							
30 0 h AE	4.95 ± 0.29^b^*	105.83 ± 6.14^b^*	171.41 ± 45.54	74.73 ± 4.25	5.06 ± 0.11	7.69 ± 0.99*	27.96 ± 5.82
60 0 h AE	6.32 ± 0.29^a^*	150.33 ± 6.14^a^*	150.99 ± 37.18	85.41 ± 4.75	5.03 ± 0.10	6.54 ± 1.57	31.46 ± 4.75
30 1 h AE	6.75 ± 0.49^a^*	92.93 ± 6.14^b^*	135.02 ± 37.18	71.39 ± 4.75	5.15 ± 0.10	10.71 ± 0.99*	38.65 ± 6.72
60 1 h AE	5.80 ± 0.70^a^*	123.95 ± 6.73^b^*	177.18 ± 37.18	83.74 ± 5.49	5.16 ± 0.11	9.74 ± 0.99*	34.02 ± 5.82
30 24 h AE	1.72 ± 0.29^bc^	67.07 ± 6.73^c^*	143.94 ± 37.18	81.16 ± 3.88	4.93 ± 0.10	11.46 ± 0.99*	28.35 ± 5.20
60 24 h AE	0.87 ± 0.29^c^*	67.32 ± 6.14^c^*	142.42 ± 40.73	84.35 ± 4.25	5.00 ± 0.10	12.10 ± 0.91*	21.69 ± 5.20
30 48 h AE	1.64 ± 0.31^bc^	56.32 ± 6.14^c^	354.36 ± 37.18*	79.20 ± 3.88	5.10 ± 0.10	11.41 ± 0.99*	32.01 ± 4.75
60 48 h AE	1.38 ± 0.29^c^	55.15 ± 6.14^c^	346.78 ± 37.18	77.19 ± 3.88	4.96 ± 0.10	11.37 ± 0.91*	29.99 ± 4.75
*p*‐Value exposure (*E*)	0.540	0.001	0.940	0.062	0.785	0.620	0.526
*p*‐Value collection (*C*)	<0.001	<0.001	<0.001	0.623	0.382	0.002	0.286
*p*‐Value interaction (*E* × *C*)	0.003	0.007	0.501	0.333	0.809	0.810	0.797

*Note*: Means followed by different letters in a column differ by Duncan's test (*p* < 0.05). Asterisk indicates significant difference compared to basal using Student's *t*‐test (*p* < 0.05). 0 h AE, immediately after exposure; 1 h AE, 1 h after exposure; 24 h AE, 24 h after exposure; 48 h AE, 48 h after exposure. Abbreviations: ALT, alanine aminotransferase; AST, aspartate aminotransferase; CLR, cholesterol; GLU, glucose; PPT, total protein; TGR, triglycerides.

Triglyceride level differed significantly among collection times but was higher than baseline only for fish of the 30‐min exposure duration at 48 h AE. Cholesterol and plasma protein did not differ significantly between exposure durations or among collection times, and there was no interaction between factors.

ALT differed significantly among collection times. Both exposure durations had higher ALT compared to baseline (except for immediately after air exposure for 60 min), with the values not returning to baseline within 48 h. No significant differences were found for AST.

### 3.2 Blood gas parameters

An effect of collection time and an interaction between exposure duration and collection time for pH were observed (Table 3). Both air exposure durations (30 and 60 min) had lower pH values compared to baseline. However, at 1 h AE, only fish exposed for 30 min still had lower blood pH than baseline, as pH increased with increasing time after exposure. Fish exposed to air for 60 min had higher pH values at 48 h AE than baseline. There were effects of exposure duration and collection time, and an interaction between factors for PvCO_2_ (Table 3).

**TABLE 3:** Values (mean ± standard error of the mean) of blood gas analysis at air exposure time (30 and 60 min) and blood collection time (0 h AE, 1 h AE, 24 h AE and 48 h AE) for *Colossoma macropomum* maintained in water at suboptimal temperature (23°C).

	pH	PvCO_2_ (mmHg)	PvO_2_ (mmHg)	sO_2_ (%)	tO_2_ (mL dL^−1^)	HCO_3_^−^ (mmol L^−1^)	StHCO_3_^−^ (mmol L^−1^)	BE (mmol L^−1^)
Means								
Basal	7.52 ± 0.04	6.30 ± 0.85	12.97 ± 4.83	61.45 ± 10.81	6.08 ± 1.19	5.97 ± 0.64	9.22 ± 0.71	−19.55 ± 1.06
Means of exposure (*E*)								
30 min	7.39 ± 0.01	8.44 ± 0.27^a^	21.29 ± 2.77	49.58 ± 3.01	4.51 ± 0.30	5.99 ± 0.16^a^	8.65 ± 0.18^a^	−20.64 ± 0.26^a^
60 min	7.42 ± 0.01	7.16 ± 0.30^b^	21.74 ± 3.16	54.51 ± 3.35	4.71 ± 0.36	5.05 ± 0.19^b^	7.98 ± 0.21^b^	−21.72 ± 0.30^b^
Means of collections (*C*)								
0 h AE	7.06 ± 0.02^c^	14.94 ± 0.37^a^	6.62 ± 3.92^b^	14.21 ± 4.56^b^	1.47 ± 0.48^c^	5.62 ± 0.23^b^	5.76 ± 0.26^b^	−24.82 ± 0.37^b^
1 h AE	7.38 ± 0.03^b^	3.87 ± 0.45^c^	30.88 ± 4.80^a^	70.27 ± 5.10^a^	5.92 ± 0.56^a^	2.79 ± 0.28^c^	6.07 ± 0.31^b^	−25.07 ± 0.46^b^
24 h AE	7.58 ± 0.02^a^	6.17 ± 0.39^b^	17.22 ± 4.11^b^	56.87 ± 4.16^a^	4.30 ± 0.43^b^	7.15 ± 0.23^a^	10.72 ± 0.26^a^	−17.28 ± 0.37^a^
48 h AE	7.58 ± 0.02^a^	6.22 ± 0.39^b^	31.33 ± 3.92^a^	66.82 ± 4.16^a^	6.48 ± 0.41^a^	6.52 ± 0.24^a^	10.71 ± 0.27^a^	−17.57 ± 0.39^a^
Means for *E* × *C*								
30 0 h AE	7.11 ± 0.03^c^*	14.58 ± 0.52^a^*	6.68 ± 5.54	14.56 ± 6.45*	1.62 ± 0.68*	5.98 ± 0.33^b^	6.25 ± 0.36^c^*	−24.10 ± 0.53^c^*
60 0 h AE	7.01 ± 0.03^c^*	15.30 ± 0.52^a^*	6.55 ± 5.54	13.86 ± 6.45*	1.32 ± 0.68*	5.27 ± 0.33^b^	5.27 ± 0.36^c^*	−25.55 ± 0.53^c^*
30 1 h AE	7.36 ± 0.03^b^*	4.43 ± 0.52^c^	28.30 ± 5.54*	67.18 ± 5.88	6.08 ± 0.56	3.08 ± 0.33^c^*	6.13 ± 0.36^c^*	−24.75 ± 0.53^c^*
60 1 h AE	7.40 ± 0.05^b^	3.30 ± 0.74^c^*	33.47 ± 7.84*	73.37 ± 8.32	5.75 ± 0.96	2.50 ± 0.46^c^*	6.00 ± 0.51^c^*	−25.40 ± 0.74^c^*
30 24 h AE	7.56 ± 0.03^a^	7.50 ± 0.57^b^	20.27 ± 5.54	54.65 ± 5.88	4.00 ± 0.61	8.50 ± 0.33^a^*	11.85 ± 0.36^a^*	−15.62 ± 0.53^a^*
60 24 h AE	7.60 ± 0.03^a^	4.85 ± 0.52^c^	14.18 ± 6.07	59.10 ± 5.88	4.60 ± 0.61	5.80 ± 0.33^b^	9.58 ± 0.36^b^	−18.95 ± 0.53^b^
30 48 h AE	7.51 ± 0.03^a^	7.25 ± 0.52^b^	29.92 ± 5.54	61.93 ± 5.88	6.33 ± 0.56	6.40 ± 0.33^b^	10.35 ± 0.36^a^	−18.08 ± 0.53^b^
60 48 h AE	7.65 ± 0.03^a^*	5.20 ± 0.57^bc^	32.75 ± 5.54	71.70 ± 5.88	7.16 ± 0.61	6.64 ± 0.36^b^	11.06 ± 0.40^a^*	−17.00 ± 0.58^b^*
*p*‐Value exposure (*E*)	0.220	0.003	0.493	0.282	0.678	<0.001	0.020	0.010
*p*‐Value collection (*C*)	<0.001	<0.001	<0.001	<0.001	<0.001	<0.001	<0.001	<0.001
*p*‐Value interaction (*E* × *C*)	0.007	0.019	0.327	0.864	0.744	<0.001	0.003	0.003

*Note*: Means followed by different letters in a column differ by Duncan's test (*p* < 0.05). Asterisk indicates significant difference compared to basal values using Student's *t*‐test (*p* < 0.05). 0 h AE, immediately after exposure; 1 h AE, 1 h after exposure; 24 h AE, 24 h after exposure; 48 h AE, 48 h after exposure. Abbreviations: BE, excess base; HCO_3_^−^, bicarbonate; pH, hydrogen potential; PvCO_2_ partial pressure of venous carbon dioxide; PvO_2_, partial pressure of venous oxygen; sO_2_, oxygen saturation; StHCO_3_^−^, standard bicarbonate; tO_2_, rate of oxygen.

The highest PvCO_2_ values were observed immediately after both air exposure durations, which were also higher than baseline. Fish exposed to air for 60 min had lower PvCO_2_ than baseline at 1 h AE. There was only an effect of collection time for PvO_2_, sO_2_ and tO_2_. PvO_2_ was higher than baseline at 1 h AE, whereas sO_2_ and tO_2_ were lower than baseline immediately after both exposure durations (Table 3).

Effects of exposure duration and collection time, and an interaction between factors, were observed for HCO_3_^−^, StHCO_3_^−^ and BE. Immediately after air exposure and at 1 h AE, fish of both exposure durations had lower HCO_3_^−^ than baseline, and those exposed for 30 min also had the highest HCO_3_^−^ levels at 24 h AE. Fish of both air exposure durations had lower StHCO_3_^−^ and BE levels compared to baseline, whereas those exposed for 60 min returned to baseline at 24 h AE, with a subsequent significant increase at 48 h AE (Table 3).

### 3.3 Blood electrolytes

An effect of collection time and an interaction between factors for K^+^ were observed (Table 4). Fish exposed to air for 30 min had lower K^+^ levels than baseline at 1 h AE, and their highest at 24 h AE. Fish exposed to air for 60 min had the lowest K^+^ levels only at 24 h AE. There was also an effect of collection time, and an interaction between factors, for Na^+^, but the responses were contrary to those for K^+^ for fish exposed to air for 30 min, that is, higher Na^+^ than baseline at 1 h AE and lowest at 24 h AE. Fish exposed to air for only 60 min had lower Na^+^ than baseline at 48 h AE. Effects of exposure duration and collection time were found for Cl^−^. An interaction between exposure duration and collection time was observed for Ca^2+^, with it being lowest at 48 h AE for fish of the 60 min exposure (Table 4).

**TABLE 4:** Values (mean ± standard error of the mean) of blood electrolytes at air exposure time (30 and 60 min) and blood collection time (0 h AE, 1 h AE, 24 h AE, 48 h AE) for *Colossoma macropomum* maintained in water at suboptimal temperature (23°C).

	K^+^ (mmol L ^−1^)	Na^+^ (mmol L^−1^)	Cl^−^ (mmol L^−1^)	Ca^2+^ (mmol L^−1^)
Means				
Basal	4.10 ± 0.24	157.50 ± 1.82	137.50 ± 3.06	0.56 ± 0.07
Means of exposure (*E*)				
30 min	4.16 ± 0.13	155.67 ± 0.95	139.42 ± 0.43^a^	0.60 ± 0.03
60 min	3.94 ± 0.14	158.04 ± 1.06	137.37 ± 0.49^b^	0.58 ± 0.04
Means of collections (*C*)				
0 h AE	4.31 ± 0.18^a^	159.25 ± 1.34^a^	139.10 ± 0.64^b^	0.58 ± 0.05
1 h AE	3.34 ± 0.22^b^	161.67 ± 1.64^a^	136.25 ± 0.72^c^	0.53 ± 0.06
24 h AE	4.38 ± 0.18^a^	154.00 ± 1.34^b^	141.90 ± 0.64^a^	0.56 ± 0.05
48 h AE	4.17 ± 0.18^a^	152.50 ± 1.34^b^	136.32 ± 0.61^c^	0.68 ± 0.05
Means for *E* × *C*				
30 0 h AE	4.23 ± 0.26^b^	157.33 ± 1.89^a^	141.40 ± 0.90	0.53 ± 0.07^a^
60 0 h AE	4.38 ± 0.26^b^	161.17 ± 1.89^a^	136.80 ± 0.90	0.64 ± 0.07^a^
30 1 h AE	2.75 ± 0.26^c^*	162.33 ± 1.89^a^*	136.83 ± 0.83	0.55 ± 0.07^a^
60 1 h AE	3.93 ± 0.37^bc^	161.00 ± 2.68^a^	135.67 ± 1.17	0.52 ± 0.10^a^
30 24 h AE	5.50 ± 0.26^a^*	148.33 ± 1.89^c^*	142.60 ± 0.90	0.69 ± 0.07^a^
60 24 h AE	3.27 ± 0.26^c^*	159.67 ± 1.89^a^	141.20 ± 0.90	0.43 ± 0.07^b^
30 48 h AE	4.17 ± 0.26^b^	154.67 ± 1.89^b^	136.83 ± 0.83	0.63 ± 0.07^a^
60 48 h AE	4.17 ± 0.26^b^	150.33 ± 1.89^b^*	135.80 ± 0.90	0.74 ± 0.07^a^
*p*‐Value exposure (*E*)	0.256	0.103	0.004	0.751
*p*‐Value collection (*C*)	0.005	<0.001	<0.001	0.245
*p*‐Value interaction (*E* × *C*)	<0.001	0.001	0.174	0.047

*Note*: Means followed by different letters in a column differ by Duncan's test (*p* < 0.05). Asterisk indicates significant difference compared to baseline using Student's *t*‐test (*p* < 0.05). 0 h AE, immediately after exposure; 1 h AE, 1 h after exposure; 24 h AE, 24 h after exposure; 48 h AE, 48 h after exposure. Abbreviations: Ca^2+^, calcium; Cl^−^, chloride; Na^+^, sodium; K^+^, potassium.

### 3.4 Oxidative status

#### 3.4.1 Reduced glutathione

**FIGURE 1:**
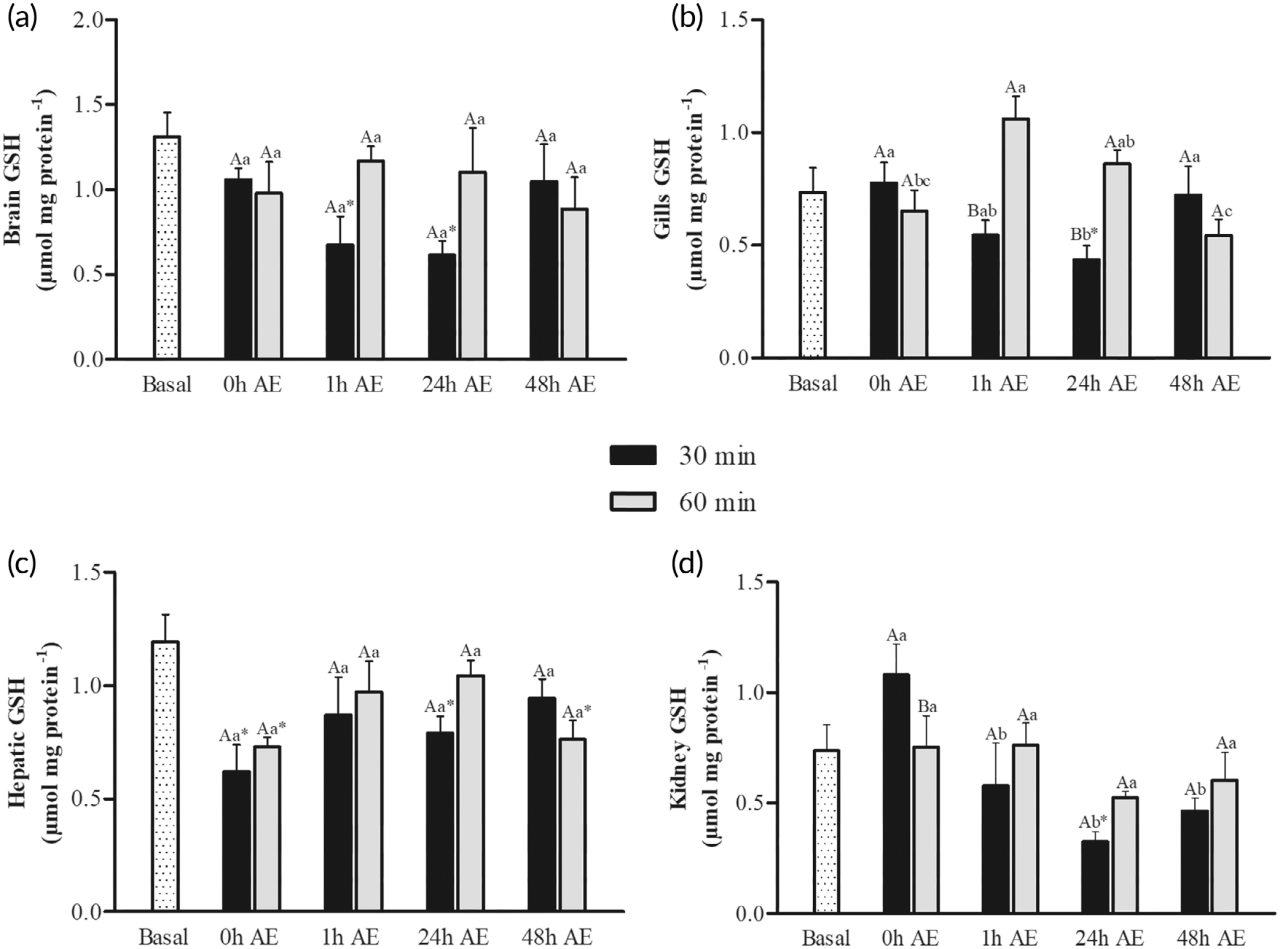
Reduced glutathione (GSH) in (a) brain, (b) gills, (c) liver and (d) kidney of *Colossoma macropomum* after exposure to air for different durations (30 and 60 min) and at different collection times (basal; 0 h AE, immediately after exposure; 1 h AE, 1 h after exposure; 24 h AE, 24 h after exposure; and 48 h AE, 48 h after exposure). Data are presented as mean ± standard error of the mean. Data were analysed using two‐way ANOVA (analysis of variance) followed by Duncan's test (*p* < 0.05). Different lowercase letters indicate a significant difference between collection times for each air exposure duration (30 and 60 min). Different capital letters indicate a significant difference between air exposure durations (30 and 60 min) at each collection time. Asterisk indicates a significant difference compared to baseline using Student's *t*‐test (*p* < 0.05).

Lower GSH levels were observed in the brain at 1 h (*p* = 0.021) and 24 h (*p* = 0.003) after 30 min of exposure (Figure 1a). Lower GSH levels were also observed in the liver immediately after exposure for 30 min (*p* = 0.007) and 60 min (*p* = 0.005), and at 24 h (*p* = 0.018) and 48 h (*p* = 0.016) for 30 and 60 min of exposures, respectively, compared to baseline (Figure 1c).

Lower GSH levels were observed in the gills after 30 min of air exposure compared to 60 min at 1 h AE and 24 h AE. Lower GSH levels were also observed after 30 min of air exposure at 24 h AE compared to baseline (*p* = 0.040).

Fish exposed to air for 60 min had higher GSH at 1 h AE compared to immediately after (*p* < 0.001) and 48 h AE (*p* = 0.017). These fish also had higher GSH than those of the 30‐min exposure at 1 h AE and 24 h AE (*p* = 0.001; Figure 1b). Fish exposed to air for 30 min had lower GSH levels in the kidneys at 24 h AE compared to baseline (*p* = 0.008), and the highest GSH levels were detected immediately after air exposure (*p* = 0.035). No significant differences in GSH levels in the kidneys were observed among the different collection times for fish exposed to air for 60 min (*p* > 0.050; Figure 1d).

#### 3.4.2 Superoxide dismutase

No SOD activity was found in the brain, gills, liver and kidneys of *C. macropomum* juveniles.

#### 3.4.3 Thiobarbituric acid reactive substances

**FIGURE 2:**
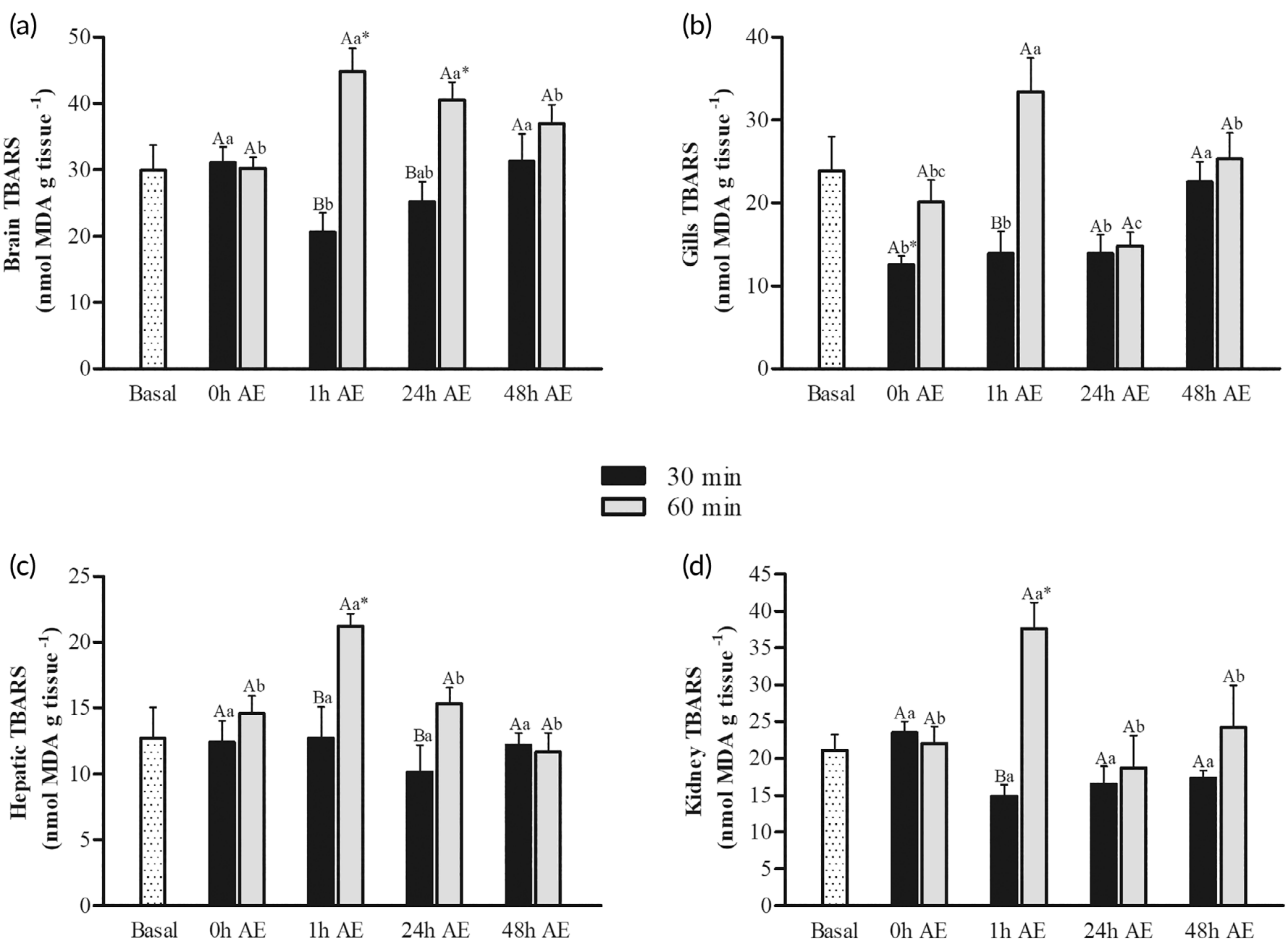
Thiobarbituric acid reactive substances (TBARS) in (a) brain, (b) gills, (c) liver and (d) kidney of *Colossoma macropomum* after air exposure for different durations (30 and 60 min) and at different collection times (basal; 0 h AE, immediately after exposure; 1 h AE, 1 h after exposure; 24 h AE, 24 h after exposure; and 48 h AE, 48 h after exposure). Data are presented as mean ± standard error of the mean. Data were analysed using two‐way ANOVA (analysis of variance) followed by Duncan's test (*p* < 0.05). Different lowercase letters indicate a significant difference between collection times for each air exposure duration (30 and 60 min). Different capital letters indicate a significant difference between air exposure durations (30 and 60 min) at each collection time. Asterisk indicates a significant difference compared to baseline using Student's *t*‐test (*p* < 0.05).

Higher levels of TBARS in the brain were observed at 1 h AE and 24 h AE for fish exposed to air for 60 min compared to those exposed to air for 30 min (*p* < 0.001 for both), as well as in relation to the other collection times and baseline (Figure 2). Fish exposed to air for 30 min had a lower level of TBARS at 1 h AE compared to immediately (*p* = 0.022) and 48 h AE (*p* = 0.023; Figure 2a). Fish exposed to air for 30 min had a lower TBARS level in the gills immediately after air exposure compared to baseline (*p* = 0.037) and the highest level compared to other times at 48 h AE (*p* < 0.001). On the contrary, fish exposed to air for 60 min had the highest TBARS level compared to other times and to fish exposed to air for 30 min at 1 h AE (*p* < 0.001; Figure 2b). The level of TBARS in the liver was higher at 1 h AE (*p* < 0.001) and 24 h AE (*p* = 0.034) for fish exposed for 60 min compared to 30 min. The level of TBARS in fish exposed to air for 60 min was higher at 1 h AE compared to the other collection times (*p* < 0.001) and baseline (*p* = 0.041; Figure 2c). The level of TBARS in the kidneys was higher at 1 h AE in fish exposed to air for 60 min compared to other collection times (*p* < 0.001), to those exposed to air for 30 min (*p* < 0.001), and to baseline (*p* = 0.026; Figure 2d).

## 4 DISCUSSION

Exposure to air can occur during the different types of handling in fish culture, such as capture, selection, biometry and transport preparation, all of which can produce different stress responses in fish. Haematological, biochemical, gasometric and oxidative parameters, therefore, can be important tools for monitoring stress in tambaqui. The present study observed alterations in haematological parameters, such as the increased haematocrit and haemoglobin concentrations immediately and 1 h after air exposure, compared to the other collection periods. According to Reid et al. (1998), these responses may be attributed to the release of catecholamines, which modulate cardiovascular and respiratory functions to maintain the levels of oxygen in the blood and a sufficient supply to the tissues, like the gills. However, higher levels of plasma lactate were also observed in these same periods, which is attributed to glucose metabolism via anaerobic glycolysis. That is, a possible deficit of body oxygen could have caused the production of lactate by white muscle, thus increasing levels in the bloodstream (Mattioli et al., 2019; Wood, 1991). Lactate levels returned to baseline levels 24 h after air exposure for both exposure durations of 30 and 60 min.

Blood glucose is another important indicator of stress in fish and can be mobilized to meet their energy demands (Carneiro & Urbinati, 2001). Increased blood glucose after stress is common, as verified in the present study, with the highest glucose level being observed immediately after the longer exposure duration (60 min). However, glucose levels returned to baseline only 48 h after air exposure for both exposure durations. These results corroborate those of other studies with juveniles of *C. macropomum* (Neves et al., 2022) and with other species, such as *Lophiosilurus alexandri* (Mattioli et al., 2019), *Sparus aurata* (Skrzynska et al., 2018), *Oreochromis niloticus* (Silva et al., 2012) and *Rachycentron canadum* (Trushenski et al., 2010), also subjected to stress by air exposure.

A higher level of blood triglycerides was observed at the later collection time after air exposure (48 h) compared to the other collection times. Although body energy reserves were not quantified in the present study, stress from air exposure and lack of food during the entire collection period may have led to the catabolism of lipids in the liver, adipose tissue and/or muscle, and an increase in plasma triglyceride levels. Similar results were found by Costas et al. (2011) in *Solea senegalensis* also subjected to air exposure.

Air exposure had no effect on plasma cholesterol levels of tambaqui. However, another study by our research group with the same species but kept at the optimal water temperature of 28°C found decreased cholesterol levels immediately and 1 h after air exposure (Neves et al., 2022). Silva et al. (2012) stated that this decrease may be due to the consumption of cholesterol that occurs during stressful situations, leading to the production of pregnenolone, a precursor substance in the synthesis of cortisol. There was also no change in the total plasma protein level, as also observed for *O. niloticus* subjected to up to 90 min of air exposure (Silva et al., 2012). However, other studies have found increased total plasma protein levels for *C. macropomum*, which is due to the mobilization of body proteins to satisfy the body's energy demand (Neves et al., 2022), or decreased circulating protein levels in *L. alexandri*, due to changes in plasma volume and an osmotic imbalance (Mattioli et al., 2019), both caused by the stress of air exposure.

Overall, higher levels of ALT were observed in the plasma of tambaqui juveniles from 1 to 48 h after 30 and 60 min of air exposure, compared to baseline, whereas there were no significant differences for AST. Both enzymes can be found in the liver and other tissues, such as the kidney, heart and skeletal muscle. Although the presence of these enzymes in the liver and other organs of *C. macropomum* juveniles was not evaluated, the increase in plasma ALT after exposure to air may be an indication of possible injuries or tissue damage, as found for *Brycon cephalus* exposed to the organophosphate agricultural pesticide Folidol (Aguiar et al., 2004), and according to Ovie et al. (2010), such changes are also attributable to stressful conditions and diseases.

Air exposure also resulted in significant changes in the gasometric parameters evaluated in the present study, especially in relation to collection times. Lower blood pH values, as well as HCO_3_^−^ and StHCO_3_^−^ concentrations, were observed immediately and 1 h after air exposure, increasing only after 24 and 48 h, in addition to decreases in PvO_2_, sO_2_ and tO_2_ immediately after air exposure. At the same time, there was an increase in PvCO_2_ concentration immediately after air exposure, with an abrupt decrease 1 h later. According to Mattioli et al. (2019), in *L. alexandri*, these responses are characteristic of respiratory and metabolic acidosis. Increased PvCO_2_ can lead to carbonic acid formation and increased HCO_3_^−^ production, leading to a decrease in blood pH. However, in the present study, the concentrations of HCO_3_^−^ and StHCO_3_^−^ decreased immediately and 1 h after air exposure, as in the study by Neves et al. (2022). Practically all these parameters returned to baseline at 24 h after air exposure, where they remained.

Plasma ion levels can be considered as stress indicators in fish, because situations such as air exposure and hypoxia can lead to increased ventilation and gill permeability, allowing increased blood flow for greater tissue oxygenation and, consequently, an increase in the loss of ions from the gills and their decrease in the bloodstream (Gonzalez & McDonald, 1992; Silva et al., 2012; Wood & Eom, 2021). This response was observed in the present study with tambaqui, in which exposure to air led to ionic imbalance in the plasma, especially for K^+^ and Na^+^. There was a significant decrease in K^+^ levels, compared to baseline, at 1 and 24 h after fish were exposed to air for 30 and 60 min, respectively, and a decrease in Na^+^ levels at 24 and 48 h after air exposure for 30 and 60 min, respectively, as well as compared to other collection times and baseline. These decreases in the concentrations of these ions corroborate the results of other studies with fish exposed to air (Neves et al., 2022) and water hypoxia (Copatti et al., 2019).

Significant differences were observed in all oxidative stress parameters measured in the present study, except for SOD. SOD is an antioxidant enzyme that can modulate the production of free radicals and thus prevent lipid peroxidation (Faria et al., 2021; Nimse & Pal, 2015). However, no SOD activity was observed in the studied tissues (brain, gills, liver and kidneys). Similarly, there was a significant increase in TBARS levels at 1 h after air exposure in all tissues of fish for 60 min. TBARS is considered a biomarker of oxidative stress and is formed as a by‐product of lipid peroxidation (Catalán et al., 2018). The lipid peroxidation process can lead to loss of selectivity in ion exchange, release of organelle content and formation of cytotoxic products, such as malonaldehyde (MDA), leading to cell death (Ferreira & Matsubara, 1997). Thus, the higher the concentration of TBARS, the greater the indication of oxidative stress and, consequently, the greater the damage to a given tissue. However, TBARS concentrations returned to baseline at 24 h after air exposure in all tissues, except for the brain, which returned only at 48 h. According to Lushchak and Bagnyukova (2007), the return to initial values may be due to the activation of antioxidant enzymes as a form of tissue protection.

GSH is a bioactive tripeptide involved in DNA and protein synthesis and the protection of cells against xenobiotic mechanisms and natural deleterious components such as free radicals and peroxides (Deponte, 2013; Locigno & Castronovo, 2001; Ming et al., 2019). The present study found different GSH responses for different air exposure durations (30 and 60 min) and collection periods for each tissue analysed. However, in general, the stress of air exposure led to a decrease in GSH activity in all tissues, especially compared to baseline, as was found by Varju et al. (2018) in the liver of *Sander lucioperca* after a long period of fasting. Madrigal et al. (2001) found GSH depletion to be due to increased lipid peroxidation in the rat brain.

## 5 CONCLUSIONS

Juveniles of *C. macropomum* were able to modify their physiological, metabolic and oxidative stress parameters when exposed to air for 30 or 60 min, reestablishing baseline conditions 48 h after exposure, even when kept in water with a suboptimal temperature of 23°C.

The findings of this study clearly and positively show how tambaqui can be resistant to common stressors in aquaculture, such as management through air exposure, and provide important information on the possibility of producing this species in places where the water temperature is considered suboptimal (23°C). With these results, more studies must be conducted on animals that are reared in water at temperatures below 23°C.

## AUTHOR CONTRIBUTIONS

**Gisele Cristina Favero:** conceptualization, formal analysis, funding acquisition, investigation, methodology, resources, validation, visualization, writing – original draft, writing – review and editing. **Luanna do Carmo Neves**: conceptualization, formal analysis, investigation, methodology, validation. **Suzane Lilian Beier:** conceptualization, investigation, methodology, validation. **Hugo Napoleão Pereira da Silva:** conceptualization, investigation, methodology, validation. **Juan Lameira Dornelles:** conceptualization, investigation, methodology, validation. **Bernardo Baldisserotto:** conceptualization, funding acquisition, investigation, methodology, validation. **Glauber David Almeida Palheta:** conceptualization, investigation, methodology, validation. **Nuno Filipe Alves Correia de Melo:** conceptualization, investigation, methodology, validation. **Ronald Kennedy Luz:** conceptualization, data curation, formal analysis, funding acquisition, investigation, methodology, project administration, resources, supervision, visualization, writing – original draft, writing – review and editing.

## FUNDING INFORMATION

This work was supported by Conselho Nacional de Desenvolvimento Científico e Tecnológico (CNPq‐Brazil‐402952/2021‐9), Coordenação de Aperfeiçoamento de Pessoal de Nível Superior (CAPES‐Brazil‐Procad 88887.200588/2018‐00) and Fundação de Amparo à Pesquisa do Estado de Minas Gerais (FAPEMIG‐Brazil). Gisele Cristina Favero, Ronald Kennedy Luz and Bernardo Baldisserotto received research grants from Conselho Nacional de Desenvolvimento Científico e Tecnológico (CNPq nos: 316901/2021‐0, 308547/2018‐7 and 301225/2017‐6, respectively).

## CONFLICT OF INTEREST STATEMENT

The authors declare that they have no known competing financial interests or personal relationships that could have influenced the work reported in this paper.

## Data Availability

The data that support the findings of this study are available from the corresponding author upon reasonable request.
